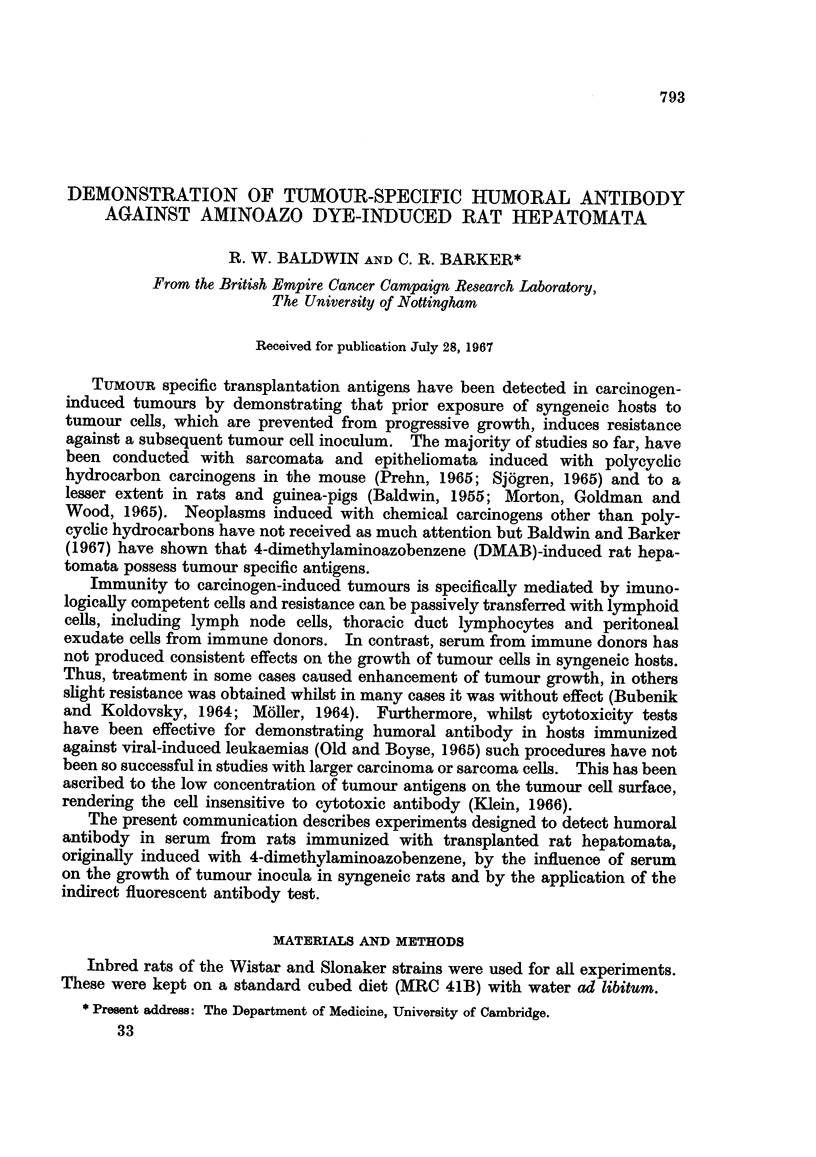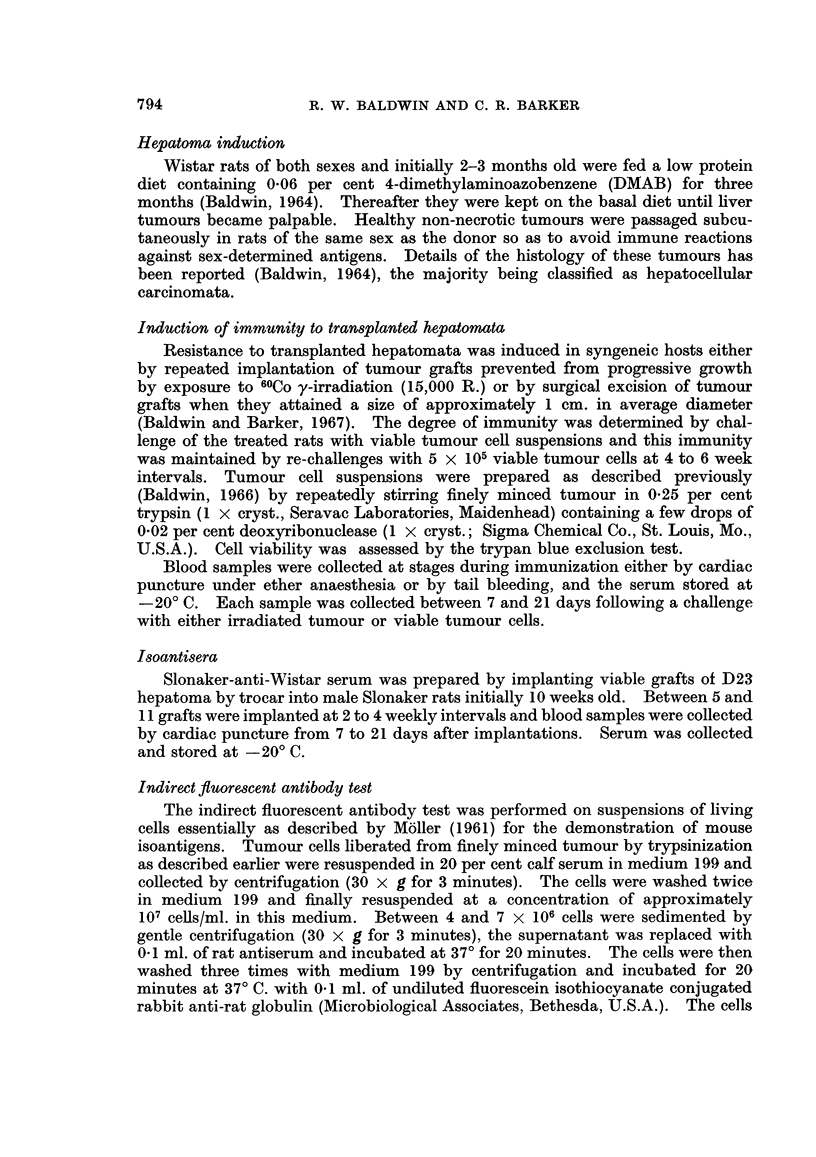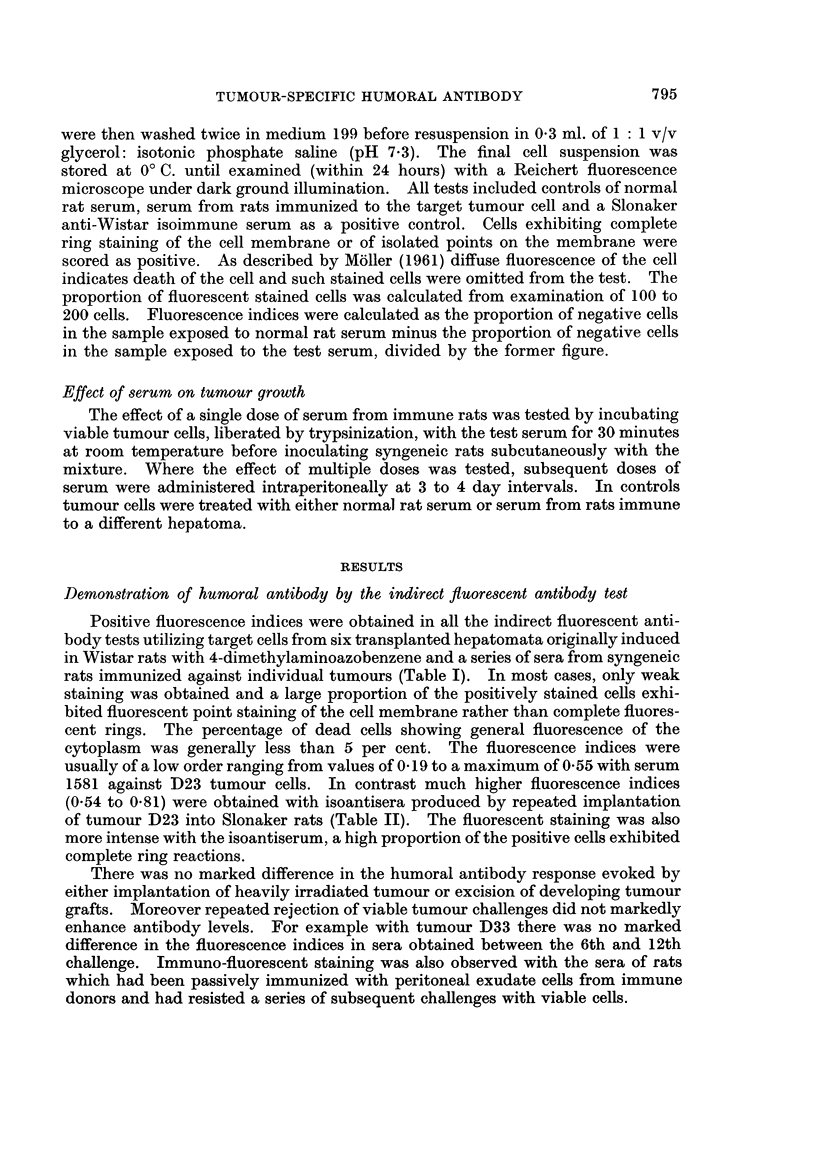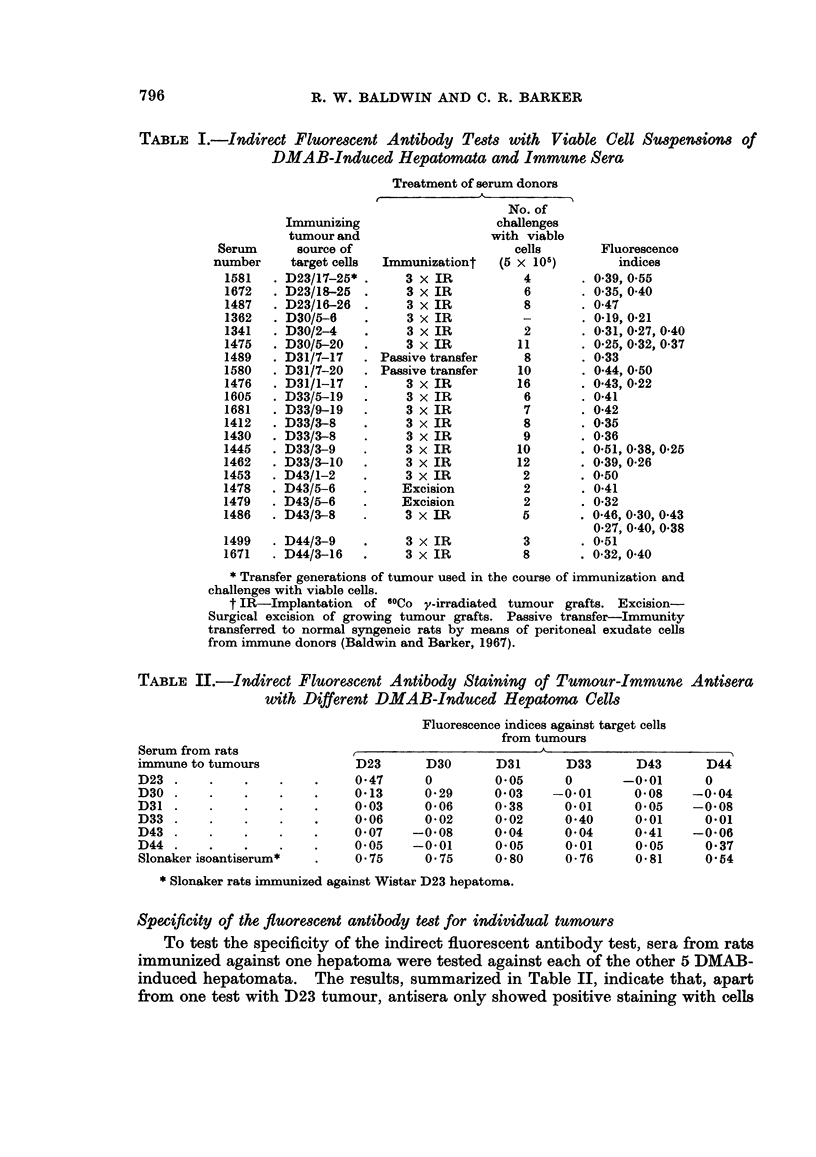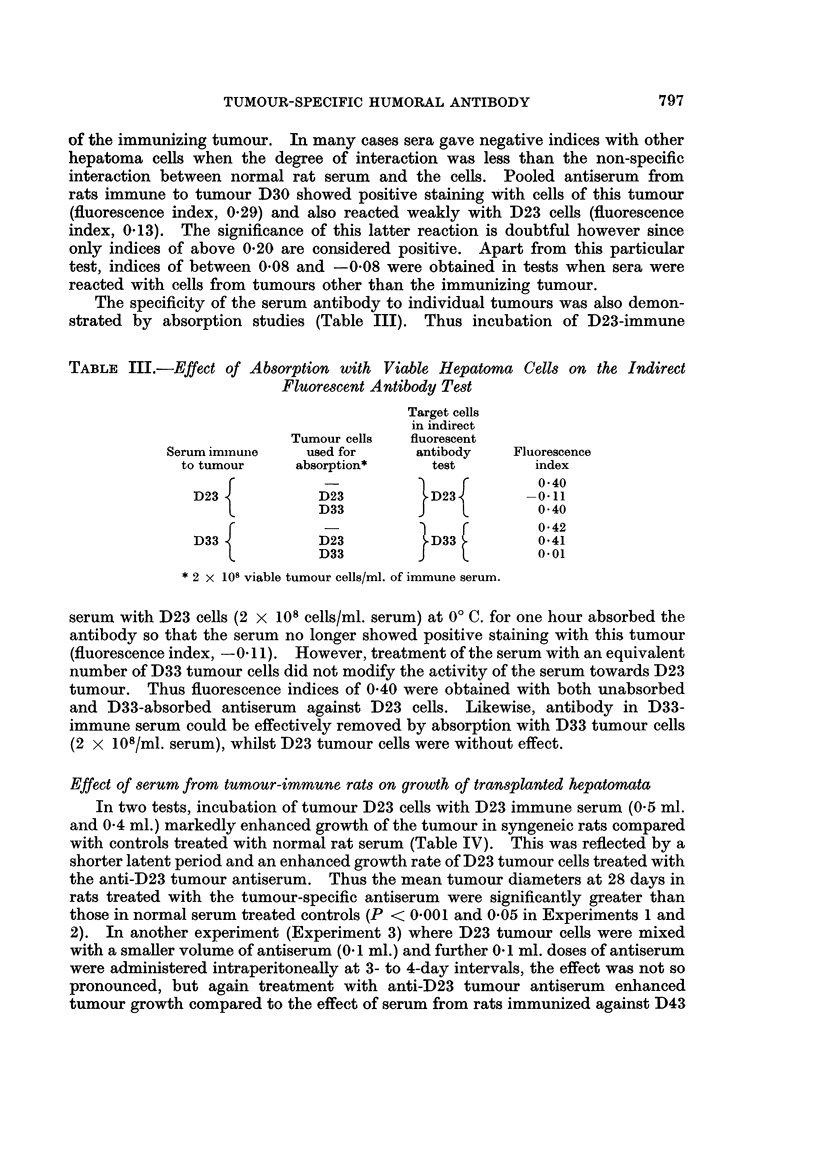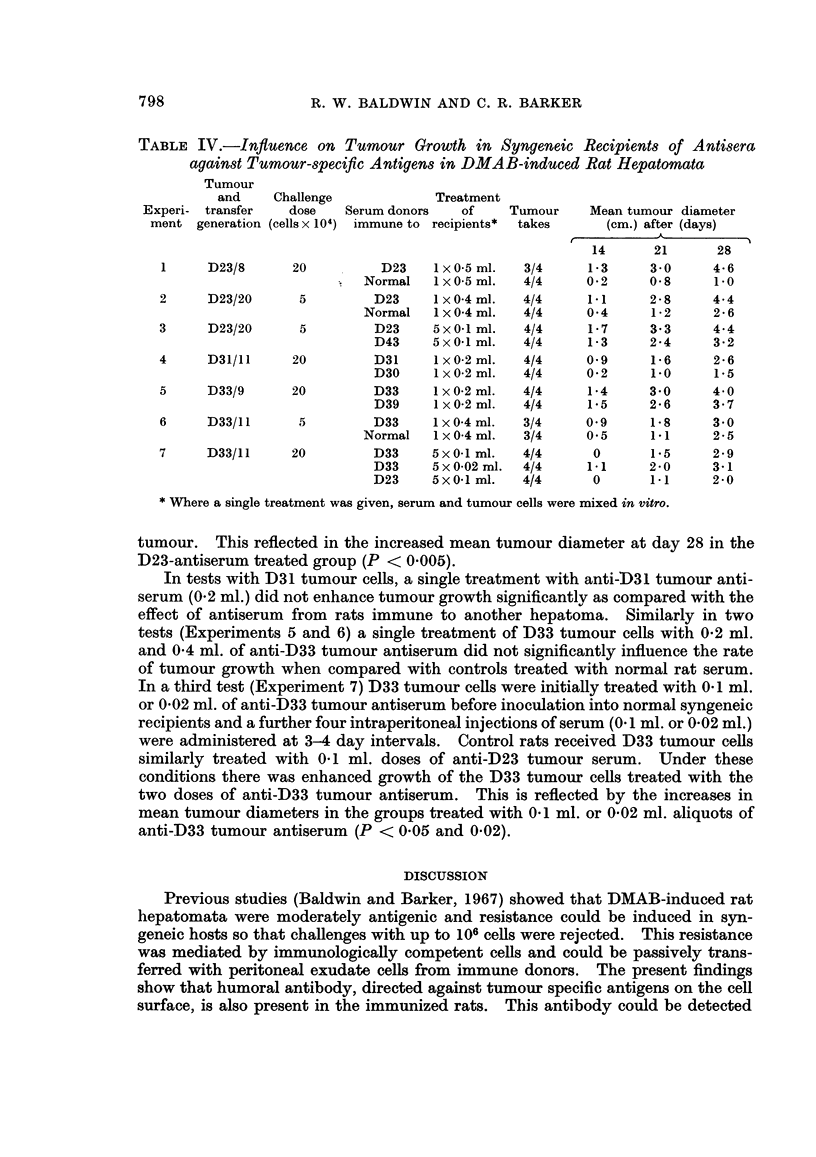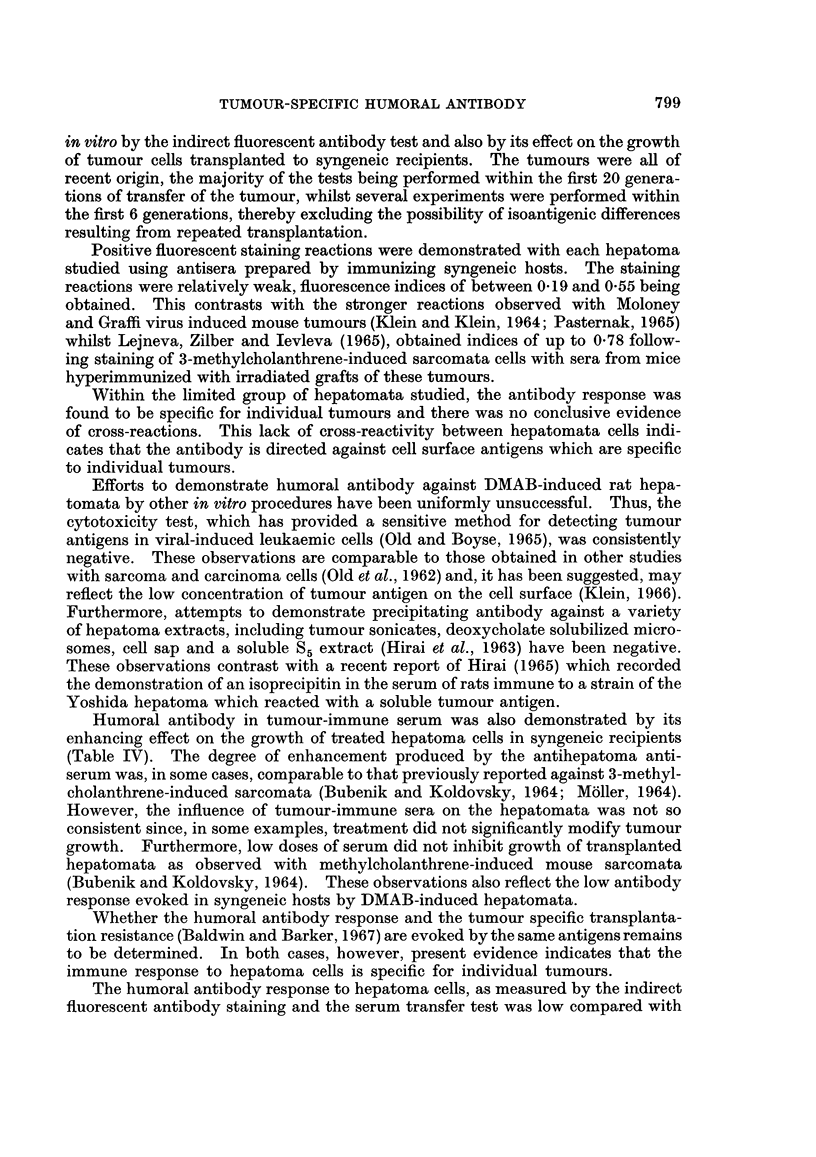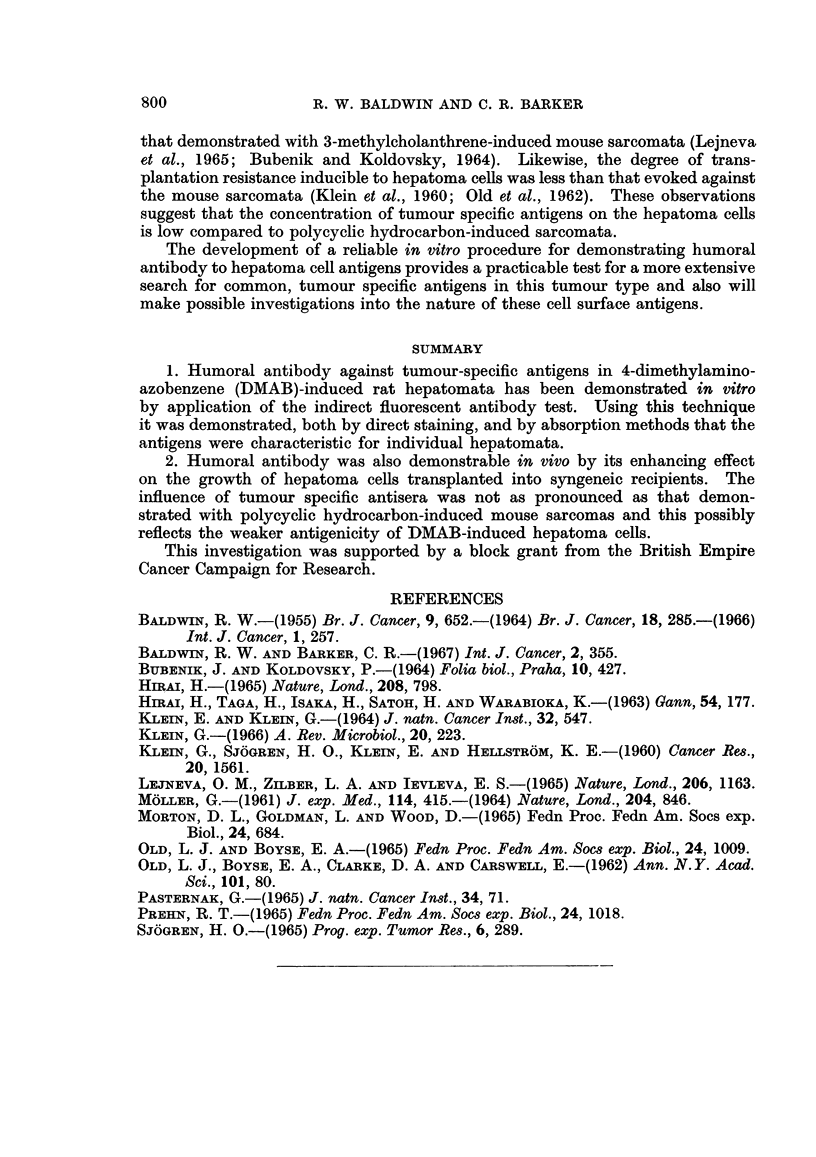# Demonstration of tumour-specific humoral antibody against aminoazo dye-induced rat hepatomata.

**DOI:** 10.1038/bjc.1967.93

**Published:** 1967-12

**Authors:** R. W. Baldwin, C. R. Barker


### DEMONSTRATION OF TUMOUR-SPECIFIC HUMORAL ANTIBODY AGAINST AMINOAZO DYE-INDUCED RAT HEPATOMATA

R. W. BALDWIN AND C. R. BARKER*

*From the British Empire Cancer Campaign Research Laboratory, The University of Nottingham*

Received for publication July 28, 1967

TUMOUR specific transplantation antigens have been detected in carcinogen-induced tumours by demonstrating that prior exposure of syngeneic hosts to tumour cells, which are prevented from progressive growth, induces resistance against a subsequent tumour cell inoculum. The majority of studies so far, have been conducted with sarcomata and epitheliomata induced with polycyclic hydrocarbon carcinogens in the mouse (Prehn, 1965; Sjögren, 1965) and to a lesser extent in rats and guinea-pigs (Baldwin, 1955; Morton, Goldman and Wood, 1965). Neoplasms induced with chemical carcinogens other than polycyclic hydrocarbons have not received as much attention but Baldwin and Barker (1967) have shown that 4-dimethylaminoazobenzene (DMAB)-induced rat hepatomata possess tumour specific antigens.

Immunity to carcinogen-induced tumours is specifically mediated by imunologically competent cells and resistance can be passively transferred with lymphoid cells, including lymph node cells, thoracic duct lymphocytes and peritoneal exudate cells from immune donors. In contrast, serum from immune donors has not produced consistent effects on the growth of tumour cells in syngeneic hosts. Thus, treatment in some cases caused enhancement of tumour growth, in others slight resistance was obtained whilst in many cases it was without effect (Bubenik and Koldovsky, 1964; Möller, 1964). Furthermore, whilst cytotoxicity tests have been effective for demonstrating humoral antibody in hosts immunized against viral-induced leukaemias (Old and Boyse, 1965) such procedures have not been so successful in studies with larger carcinoma or sarcoma cells. This has been ascribed to the low concentration of tumour antigens on the tumour cell surface, rendering the cell insensitive to cytotoxic antibody (Klein, 1966).

The present communication describes experiments designed to detect humoral antibody in serum from rats immunized with transplanted rat hepatomata, originally induced with 4-dimethylaminoazobenzene, by the influence of serum on the growth of tumour inocula in syngeneic rats and by the application of the indirect fluorescent antibody test.

### MATERIALS AND METHODS

Inbred rats of the Wistar and Slonaker strains were used for all experiments. These were kept on a standard cubed diet (MRC 41B) with water *ad libitum*.

* Present address: The Department of Medicine, University of Cambridge.

*Hepatoma induction*

Wistar rats of both sexes and initially 2–3 months old were fed a low protein diet containing 0·06 per cent 4-dimethylaminoazobenzene (DMAB) for three months (Baldwin, 1964). Thereafter they were kept on the basal diet until liver tumours became palpable. Healthy non-necrotic tumours were passaged subcutaneously in rats of the same sex as the donor so as to avoid immune reactions against sex-determined antigens. Details of the histology of these tumours has been reported (Baldwin, 1964), the majority being classified as hepatocellular carcinomata.

*Induction of immunity to transplanted hepatomata*

Resistance to transplanted hepatomata was induced in syngeneic hosts either by repeated implantation of tumour grafts prevented from progressive growth by exposure to $^{60}$Co $\gamma$-irradiation (15,000 R.) or by surgical excision of tumour grafts when they attained a size of approximately 1 cm. in average diameter (Baldwin and Barker, 1967). The degree of immunity was determined by challenge of the treated rats with viable tumour cell suspensions and this immunity was maintained by re-challenges with $5 \times 10^5$ viable tumour cells at 4 to 6 week intervals. Tumour cell suspensions were prepared as described previously (Baldwin, 1966) by repeatedly stirring finely minced tumour in 0·25 per cent trypsin (1 × cryst., Seravac Laboratories, Maidenhead) containing a few drops of 0·02 per cent deoxyribonuclease (1 × cryst.; Sigma Chemical Co., St. Louis, Mo., U.S.A.). Cell viability was assessed by the trypan blue exclusion test.

Blood samples were collected at stages during immunization either by cardiac puncture under ether anaesthesia or by tail bleeding, and the serum stored at −20° C. Each sample was collected between 7 and 21 days following a challenge with either irradiated tumour or viable tumour cells.

*Isoantisera*

Slonaker-anti-Wistar serum was prepared by implanting viable grafts of D23 hepatoma by trocar into male Slonaker rats initially 10 weeks old. Between 5 and 11 grafts were implanted at 2 to 4 weekly intervals and blood samples were collected by cardiac puncture from 7 to 21 days after implantations. Serum was collected and stored at −20° C.

*Indirect fluorescent antibody test*

The indirect fluorescent antibody test was performed on suspensions of living cells essentially as described by Möller (1961) for the demonstration of mouse isoantigens. Tumour cells liberated from finely minced tumour by trypsinization as described earlier were resuspended in 20 per cent calf serum in medium 199 and collected by centrifugation (30 × *g* for 3 minutes). The cells were washed twice in medium 199 and finally resuspended at a concentration of approximately $10^7$ cells/ml. in this medium. Between 4 and $7 \times 10^6$ cells were sedimented by gentle centrifugation (30 × *g* for 3 minutes), the supernatant was replaced with 0·1 ml. of rat antiserum and incubated at 37° for 20 minutes. The cells were then washed three times with medium 199 by centrifugation and incubated for 20 minutes at 37° C. with 0·1 ml. of undiluted fluorescein isothiocyanate conjugated rabbit anti-rat globulin (Microbiological Associates, Bethesda, U.S.A.). The cells were then washed twice in medium 199 before resuspension in 0·3 ml. of 1 : 1 v/v glycerol: isotonic phosphate saline (pH 7·3). The final cell suspension was stored at 0° C. until examined (within 24 hours) with a Reichert fluorescence microscope under dark ground illumination. All tests included controls of normal rat serum, serum from rats immunized to the target tumour cell and a Slonaker anti-Wistar isoimmune serum as a positive control. Cells exhibiting complete ring staining of the cell membrane or of isolated points on the membrane were scored as positive. As described by Möller (1961) diffuse fluorescence of the cell indicates death of the cell and such stained cells were omitted from the test. The proportion of fluorescent stained cells was calculated from examination of 100 to 200 cells. Fluorescence indices were calculated as the proportion of negative cells in the sample exposed to normal rat serum minus the proportion of negative cells in the sample exposed to the test serum, divided by the former figure.

*Effect of serum on tumour growth*

The effect of a single dose of serum from immune rats was tested by incubating viable tumour cells, liberated by trypsinization, with the test serum for 30 minutes at room temperature before inoculating syngeneic rats subcutaneously with the mixture. Where the effect of multiple doses was tested, subsequent doses of serum were administered intraperitoneally at 3 to 4 day intervals. In controls tumour cells were treated with either normal rat serum or serum from rats immune to a different hepatoma.

### RESULTS

*Demonstration of humoral antibody by the indirect fluorescent antibody test*

Positive fluorescence indices were obtained in all the indirect fluorescent antibody tests utilizing target cells from six transplanted hepatomata originally induced in Wistar rats with 4-dimethylaminoazobenzene and a series of sera from syngeneic rats immunized against individual tumours (Table I). In most cases, only weak staining was obtained and a large proportion of the positively stained cells exhibited fluorescent point staining of the cell membrane rather than complete fluorescent rings. The percentage of dead cells showing general fluorescence of the cytoplasm was generally less than 5 per cent. The fluorescence indices were usually of a low order ranging from values of 0·19 to a maximum of 0·55 with serum 1581 against D23 tumour cells. In contrast much higher fluorescence indices (0·54 to 0·81) were obtained with isoantisera produced by repeated implantation of tumour D23 into Slonaker rats (Table II). The fluorescent staining was also more intense with the isoantiserum, a high proportion of the positive cells exhibited complete ring reactions.

There was no marked difference in the humoral antibody response evoked by either implantation of heavily irradiated tumour or excision of developing tumour grafts. Moreover repeated rejection of viable tumour challenges did not markedly enhance antibody levels. For example with tumour D33 there was no marked difference in the fluorescence indices in sera obtained between the 6th and 12th challenge. Immuno-fluorescent staining was also observed with the sera of rats which had been passively immunized with peritoneal exudate cells from immune donors and had resisted a series of subsequent challenges with viable cells.

TABLE I.—*Indirect Fluorescent Antibody Tests with Viable Cell Suspensions of DMAB-Induced Hepatomata and Immune Sera*

| Serum number | Immunizing tumour and source of target cells | Treatment of serum donors: Immunization† | Treatment of serum donors: No. of challenges with viable cells ($5 \times 10^5$) | Fluorescence indices |
|---|---|---|---|---|
| 1581 | D23/17–25* | 3 × IR | 4 | 0·39, 0·55 |
| 1672 | D23/18–25 | 3 × IR | 6 | 0·35, 0·40 |
| 1487 | D23/16–26 | 3 × IR | 8 | 0·47 |
| 1362 | D30/5–6 | 3 × IR | – | 0·19, 0·21 |
| 1341 | D30/2–4 | 3 × IR | 2 | 0·31, 0·27, 0·40 |
| 1475 | D30/5–20 | 3 × IR | 11 | 0·25, 0·32, 0·37 |
| 1489 | D31/7–17 | Passive transfer | 8 | 0·33 |
| 1580 | D31/7–20 | Passive transfer | 10 | 0·44, 0·50 |
| 1476 | D31/1–17 | 3 × IR | 16 | 0·43, 0·22 |
| 1605 | D33/5–19 | 3 × IR | 6 | 0·41 |
| 1681 | D33/9–19 | 3 × IR | 7 | 0·42 |
| 1412 | D33/3–8 | 3 × IR | 8 | 0·35 |
| 1430 | D33/3–8 | 3 × IR | 9 | 0·36 |
| 1445 | D33/3–9 | 3 × IR | 10 | 0·51, 0·38, 0·25 |
| 1462 | D33/3–10 | 3 × IR | 12 | 0·39, 0·26 |
| 1453 | D43/1–2 | 3 × IR | 2 | 0·50 |
| 1478 | D43/5–6 | Excision | 2 | 0·41 |
| 1479 | D43/5–6 | Excision | 2 | 0·32 |
| 1486 | D43/3–8 | 3 × IR | 5 | 0·46, 0·30, 0·43 0·27, 0·40, 0·38 |
| 1499 | D44/3–9 | 3 × IR | 3 | 0·51 |
| 1671 | D44/3–16 | 3 × IR | 8 | 0·32, 0·40 |

* Transfer generations of tumour used in the course of immunization and challenges with viable cells.

† IR—Implantation of $^{60}Co$ $\gamma$-irradiated tumour grafts. Excision—Surgical excision of growing tumour grafts. Passive transfer—Immunity transferred to normal syngeneic rats by means of peritoneal exudate cells from immune donors (Baldwin and Barker, 1967).

TABLE II.—*Indirect Fluorescent Antibody Staining of Tumour-Immune Antisera with Different DMAB-Induced Hepatoma Cells*

| Serum from rats immune to tumours | Fluorescence indices against target cells from tumours: D23 | D30 | D31 | D33 | D43 | D44 |
|---|---|---|---|---|---|---|
| D23 | 0·47 | 0 | 0·05 | 0 | −0·01 | 0 |
| D30 | 0·13 | 0·29 | 0·03 | −0·01 | 0·08 | −0·04 |
| D31 | 0·03 | 0·06 | 0·38 | 0·01 | 0·05 | −0·08 |
| D33 | 0·06 | 0·02 | 0·02 | 0·40 | 0·01 | 0·01 |
| D43 | 0·07 | −0·08 | 0·04 | 0·04 | 0·41 | −0·06 |
| D44 | 0·05 | −0·01 | 0·05 | 0·01 | 0·05 | 0·37 |
| Slonaker isoantiserum* | 0·75 | 0·75 | 0·80 | 0·76 | 0·81 | 0·54 |

* Slonaker rats immunized against Wistar D23 hepatoma.

### *Specificity of the fluorescent antibody test for individual tumours*

To test the specificity of the indirect fluorescent antibody test, sera from rats immunized against one hepatoma were tested against each of the other 5 DMAB-induced hepatomata. The results, summarized in Table II, indicate that, apart from one test with D23 tumour, antisera only showed positive staining with cells of the immunizing tumour. In many cases sera gave negative indices with other hepatoma cells when the degree of interaction was less than the non-specific interaction between normal rat serum and the cells. Pooled antiserum from rats immune to tumour D30 showed positive staining with cells of this tumour (fluorescence index, 0·29) and also reacted weakly with D23 cells (fluorescence index, 0·13). The significance of this latter reaction is doubtful however since only indices of above 0·20 are considered positive. Apart from this particular test, indices of between 0·08 and —0·08 were obtained in tests when sera were reacted with cells from tumours other than the immunizing tumour.

The specificity of the serum antibody to individual tumours was also demonstrated by absorption studies (Table III). Thus incubation of D23-immune

TABLE III.—*Effect of Absorption with Viable Hepatoma Cells on the Indirect Fluorescent Antibody Test*

| Serum immune to tumour | Tumour cells used for absorption* | Target cells in indirect fluorescent antibody test | Fluorescence index |
|---|---|---|---|
| D23 | — | D23 | 0·40 |
| | D23 | | −0·11 |
| | D33 | | 0·40 |
| D33 | — | D33 | 0·42 |
| | D23 | | 0·41 |
| | D33 | | 0·01 |

* 2 × $10^8$ viable tumour cells/ml. of immune serum.

serum with D23 cells (2 × $10^8$ cells/ml. serum) at 0° C. for one hour absorbed the antibody so that the serum no longer showed positive staining with this tumour (fluorescence index, —0·11). However, treatment of the serum with an equivalent number of D33 tumour cells did not modify the activity of the serum towards D23 tumour. Thus fluorescence indices of 0·40 were obtained with both unabsorbed and D33-absorbed antiserum against D23 cells. Likewise, antibody in D33-immune serum could be effectively removed by absorption with D33 tumour cells (2 × $10^8$/ml. serum), whilst D23 tumour cells were without effect.

*Effect of serum from tumour-immune rats on growth of transplanted hepatomata*

In two tests, incubation of tumour D23 cells with D23 immune serum (0·5 ml. and 0·4 ml.) markedly enhanced growth of the tumour in syngeneic rats compared with controls treated with normal rat serum (Table IV). This was reflected by a shorter latent period and an enhanced growth rate of D23 tumour cells treated with the anti-D23 tumour antiserum. Thus the mean tumour diameters at 28 days in rats treated with the tumour-specific antiserum were significantly greater than those in normal serum treated controls ($P < 0{\cdot}001$ and 0·05 in Experiments 1 and 2). In another experiment (Experiment 3) where D23 tumour cells were mixed with a smaller volume of antiserum (0·1 ml.) and further 0·1 ml. doses of antiserum were administered intraperitoneally at 3- to 4-day intervals, the effect was not so pronounced, but again treatment with anti-D23 tumour antiserum enhanced tumour growth compared to the effect of serum from rats immunized against D43

TABLE IV.—*Influence on Tumour Growth in Syngeneic Recipients of Antisera against Tumour-specific Antigens in DMAB-induced Rat Hepatomata*

| Experiment | Tumour and transfer generation | Challenge dose (cells × $10^4$) | Serum donors immune to | Treatment of recipients* | Tumour takes | Mean tumour diameter (cm.) after (days) | | |
|---|---|---|---|---|---|---|---|---|
| | | | | | | 14 | 21 | 28 |
| 1 | D23/8 | 20 | D23 | 1 × 0·5 ml. | 3/4 | 1·3 | 3·0 | 4·6 |
| | | | Normal | 1 × 0·5 ml. | 4/4 | 0·2 | 0·8 | 1·0 |
| 2 | D23/20 | 5 | D23 | 1 × 0·4 ml. | 4/4 | 1·1 | 2·8 | 4·4 |
| | | | Normal | 1 × 0·4 ml. | 4/4 | 0·4 | 1·2 | 2·6 |
| 3 | D23/20 | 5 | D23 | 5 × 0·1 ml. | 4/4 | 1·7 | 3·3 | 4·4 |
| | | | D43 | 5 × 0·1 ml. | 4/4 | 1·3 | 2·4 | 3·2 |
| 4 | D31/11 | 20 | D31 | 1 × 0·2 ml. | 4/4 | 0·9 | 1·6 | 2·6 |
| | | | D30 | 1 × 0·2 ml. | 4/4 | 0·2 | 1·0 | 1·5 |
| 5 | D33/9 | 20 | D33 | 1 × 0·2 ml. | 4/4 | 1·4 | 3·0 | 4·0 |
| | | | D39 | 1 × 0·2 ml. | 4/4 | 1·5 | 2·6 | 3·7 |
| 6 | D33/11 | 5 | D33 | 1 × 0·4 ml. | 3/4 | 0·9 | 1·8 | 3·0 |
| | | | Normal | 1 × 0·4 ml. | 3/4 | 0·5 | 1·1 | 2·5 |
| 7 | D33/11 | 20 | D33 | 5 × 0·1 ml. | 4/4 | 0 | 1·5 | 2·9 |
| | | | D33 | 5 × 0·02 ml. | 4/4 | 1·1 | 2·0 | 3·1 |
| | | | D23 | 5 × 0·1 ml. | 4/4 | 0 | 1·1 | 2·0 |

* Where a single treatment was given, serum and tumour cells were mixed *in vitro*.

tumour. This reflected in the increased mean tumour diameter at day 28 in the D23-antiserum treated group ($P < 0{\cdot}005$).

In tests with D31 tumour cells, a single treatment with anti-D31 tumour antiserum (0·2 ml.) did not enhance tumour growth significantly as compared with the effect of antiserum from rats immune to another hepatoma. Similarly in two tests (Experiments 5 and 6) a single treatment of D33 tumour cells with 0·2 ml. and 0·4 ml. of anti-D33 tumour antiserum did not significantly influence the rate of tumour growth when compared with controls treated with normal rat serum. In a third test (Experiment 7) D33 tumour cells were initially treated with 0·1 ml. or 0·02 ml. of anti-D33 tumour antiserum before inoculation into normal syngeneic recipients and a further four intraperitoneal injections of serum (0·1 ml. or 0·02 ml.) were administered at 3–4 day intervals. Control rats received D33 tumour cells similarly treated with 0·1 ml. doses of anti-D23 tumour serum. Under these conditions there was enhanced growth of the D33 tumour cells treated with the two doses of anti-D33 tumour antiserum. This is reflected by the increases in mean tumour diameters in the groups treated with 0·1 ml. or 0·02 ml. aliquots of anti-D33 tumour antiserum ($P < 0{\cdot}05$ and 0·02).

### DISCUSSION

Previous studies (Baldwin and Barker, 1967) showed that DMAB-induced rat hepatomata were moderately antigenic and resistance could be induced in syngeneic hosts so that challenges with up to $10^6$ cells were rejected. This resistance was mediated by immunologically competent cells and could be passively transferred with peritoneal exudate cells from immune donors. The present findings show that humoral antibody, directed against tumour specific antigens on the cell surface, is also present in the immunized rats. This antibody could be detected

*in vitro* by the indirect fluorescent antibody test and also by its effect on the growth of tumour cells transplanted to syngeneic recipients. The tumours were all of recent origin, the majority of the tests being performed within the first 20 generations of transfer of the tumour, whilst several experiments were performed within the first 6 generations, thereby excluding the possibility of isoantigenic differences resulting from repeated transplantation.

Positive fluorescent staining reactions were demonstrated with each hepatoma studied using antisera prepared by immunizing syngeneic hosts. The staining reactions were relatively weak, fluorescence indices of between 0·19 and 0·55 being obtained. This contrasts with the stronger reactions observed with Moloney and Graffi virus induced mouse tumours (Klein and Klein, 1964; Pasternak, 1965) whilst Lejneva, Zilber and Ievleva (1965), obtained indices of up to 0·78 following staining of 3-methylcholanthrene-induced sarcomata cells with sera from mice hyperimmunized with irradiated grafts of these tumours.

Within the limited group of hepatomata studied, the antibody response was found to be specific for individual tumours and there was no conclusive evidence of cross-reactions. This lack of cross-reactivity between hepatomata cells indicates that the antibody is directed against cell surface antigens which are specific to individual tumours.

Efforts to demonstrate humoral antibody against DMAB-induced rat hepatomata by other *in vitro* procedures have been uniformly unsuccessful. Thus, the cytotoxicity test, which has provided a sensitive method for detecting tumour antigens in viral-induced leukaemic cells (Old and Boyse, 1965), was consistently negative. These observations are comparable to those obtained in other studies with sarcoma and carcinoma cells (Old *et al.*, 1962) and, it has been suggested, may reflect the low concentration of tumour antigen on the cell surface (Klein, 1966). Furthermore, attempts to demonstrate precipitating antibody against a variety of hepatoma extracts, including tumour sonicates, deoxycholate solubilized microsomes, cell sap and a soluble $S_5$ extract (Hirai *et al.*, 1963) have been negative. These observations contrast with a recent report of Hirai (1965) which recorded the demonstration of an isoprecipitin in the serum of rats immune to a strain of the Yoshida hepatoma which reacted with a soluble tumour antigen.

Humoral antibody in tumour-immune serum was also demonstrated by its enhancing effect on the growth of treated hepatoma cells in syngeneic recipients (Table IV). The degree of enhancement produced by the antihepatoma antiserum was, in some cases, comparable to that previously reported against 3-methylcholanthrene-induced sarcomata (Bubenik and Koldovsky, 1964; Möller, 1964). However, the influence of tumour-immune sera on the hepatomata was not so consistent since, in some examples, treatment did not significantly modify tumour growth. Furthermore, low doses of serum did not inhibit growth of transplanted hepatomata as observed with methylcholanthrene-induced mouse sarcomata (Bubenik and Koldovsky, 1964). These observations also reflect the low antibody response evoked in syngeneic hosts by DMAB-induced hepatomata.

Whether the humoral antibody response and the tumour specific transplantation resistance (Baldwin and Barker, 1967) are evoked by the same antigens remains to be determined. In both cases, however, present evidence indicates that the immune response to hepatoma cells is specific for individual tumours.

The humoral antibody response to hepatoma cells, as measured by the indirect fluorescent antibody staining and the serum transfer test was low compared with that demonstrated with 3-methylcholanthrene-induced mouse sarcomata (Lejneva *et al.*, 1965; Bubenik and Koldovsky, 1964). Likewise, the degree of transplantation resistance inducible to hepatoma cells was less than that evoked against the mouse sarcomata (Klein *et al.*, 1960; Old *et al.*, 1962). These observations suggest that the concentration of tumour specific antigens on the hepatoma cells is low compared to polycyclic hydrocarbon-induced sarcomata.

The development of a reliable *in vitro* procedure for demonstrating humoral antibody to hepatoma cell antigens provides a practicable test for a more extensive search for common, tumour specific antigens in this tumour type and also will make possible investigations into the nature of these cell surface antigens.

SUMMARY

1. Humoral antibody against tumour-specific antigens in 4-dimethylamino-azobenzene (DMAB)-induced rat hepatomata has been demonstrated *in vitro* by application of the indirect fluorescent antibody test. Using this technique it was demonstrated, both by direct staining, and by absorption methods that the antigens were characteristic for individual hepatomata.

2. Humoral antibody was also demonstrable *in vivo* by its enhancing effect on the growth of hepatoma cells transplanted into syngeneic recipients. The influence of tumour specific antisera was not as pronounced as that demonstrated with polycyclic hydrocarbon-induced mouse sarcomas and this possibly reflects the weaker antigenicity of DMAB-induced hepatoma cells.

This investigation was supported by a block grant from the British Empire Cancer Campaign for Research.

REFERENCES

BALDWIN, R. W.—(1955) *Br. J. Cancer*, **9**, 652.—(1964) *Br. J. Cancer*, **18**, 285.—(1966) *Int. J. Cancer*, **1**, 257.
BALDWIN, R. W. AND BARKER, C. R.—(1967) *Int. J. Cancer*, **2**, 355.
BUBENIK, J. AND KOLDOVSKY, P.—(1964) *Folia biol., Praha*, **10**, 427.
HIRAI, H.—(1965) *Nature, Lond.*, **208**, 798.
HIRAI, H., TAGA, H., ISAKA, H., SATOH, H. AND WARABIOKA, K.—(1963) *Gann*, **54**, 177.
KLEIN, E. AND KLEIN, G.—(1964) *J. natn. Cancer Inst.*, **32**, 547.
KLEIN, G.—(1966) *A. Rev. Microbiol.*, **20**, 223.
KLEIN, G., SJÖGREN, H. O., KLEIN, E. AND HELLSTRÖM, K. E.—(1960) *Cancer Res.*, **20**, 1561.
LEJNEVA, O. M., ZILBER, L. A. AND IEVLEVA, E. S.—(1965) *Nature, Lond.*, **206**, 1163.
MÖLLER, G.—(1961) *J. exp. Med.*, **114**, 415.—(1964) *Nature, Lond.*, **204**, 846.
MORTON, D. L., GOLDMAN, L. AND WOOD, D.—(1965) Fedn Proc. Fedn Am. Socs exp. Biol., **24**, 684.
OLD, L. J. AND BOYSE, E. A.—(1965) *Fedn Proc. Fedn Am. Socs exp. Biol.*, **24**, 1009.
OLD, L. J., BOYSE, E. A., CLARKE, D. A. AND CARSWELL, E.—(1962) *Ann. N.Y. Acad. Sci.*, **101**, 80.
PASTERNAK, G.—(1965) *J. natn. Cancer Inst.*, **34**, 71.
PREHN, R. T.—(1965) *Fedn Proc. Fedn Am. Socs exp. Biol.*, **24**, 1018.
SJÖGREN, H. O.—(1965) *Prog. exp. Tumor Res.*, **6**, 289.